# Comparative Adsorption of Porcine Reproductive and Respiratory Syndrome Virus Strains to Minnesota Soils

**DOI:** 10.3390/v17010058

**Published:** 2025-01-01

**Authors:** Joaquin Alvarez-Norambuena, Angie Quinonez-Munoz, Cesar A. Corzo, Sagar M. Goyal

**Affiliations:** Department of Veterinary Population Medicine, University of Minnesota, Saint Paul, MN 55108, USA; jalvare@umn.edu (J.A.-N.); angieqm2509@gmail.com (A.Q.-M.); corzo@umn.edu (C.A.C.)

**Keywords:** PRRS, percolation, swine, transmission

## Abstract

Porcine reproductive and respiratory syndrome (PRRS) is an endemic disease affecting the swine industry. The disease is caused by the PRRS virus (PRRSV). Despite extensive biosecurity and control measures, the persistence and seasonality of the virus have raised questions about the virus’s environmental dynamics during the fall season when the yearly epidemic onset begins and when crop harvesting and manure incorporation into the field occur. Therefore, this study aimed to assess the potential for PRRSV to percolate through different soil types, simulating conditions that could lead to groundwater contamination which could represent a risk of herd introduction. An experimental soil column model was used to mimic field conditions. Three PRRSV-2 strains were tested across thirteen Minnesota soils with different physical and chemical characteristics. The findings revealed that PRRSV can percolate through all soil types and that the amount of virus percolated decreases with increased amounts of soil. These results suggest that PRRSV can percolate through different soil types. Further investigations should be undertaken to determine the associated implications for swine health and biosecurity measures.

## 1. Introduction

Porcine reproductive and respiratory syndrome (PRRS) is an endemic disease first described in the United States of America (USA) in the 1980s, after which it spread throughout the swine industry in the country [1,2]. The disease, caused by the PRRS virus (PRRSV) causes high economic losses due to poor reproductive performance, high mortality in growing pigs, and health-associated costs, including vaccines, antibiotics, and veterinary services [3], in the USA it is primarily associated with PRRSV-2 [4]. PRRSV is shed in the semen, saliva, urine, and feces of infected animals leading to the contamination of the environment and fomites. It is known that fomites are a main source of the virus, requiring biosecurity measures such as the regular cleaning and disinfection of potential virus vehicles including transportation trucks and farm supplies [5,6,7].

Over time, the virus has demonstrated complex epidemiological dynamics, with the emergence of new variants that negatively affect swine populations [8]. Recently, data collected by the Morrison Swine Health Monitoring Project (MSHMP) revealed a consistent seasonal and temporal–spatial component in the onset of PRRS cases between October and December in U.S. swine breeding herds [9]. However, the epidemiology of seasonal outbreaks caused by multiple variants of the same virus is not fully understood. Despite critical investments in biosecurity, the virus continues to invade breeding and growing pig herds. The seasonal occurrence of PRRS coincides with crop harvesting and manure management procedures, especially in the Midwest. The role of these two events in the epidemiology of the virus is not well understood [9,10]. PRRSV has been detected through RT-PCR in 9% of the pig-barn manure pits assessed under field conditions. Furthermore, under experimental conditions, viral RNA was detected in slurry for 4 weeks post-inoculation [11].

Given that the PRRS epidemic begins during the fall and crop harvesting and manure incorporation occur simultaneously, understanding whether the virus in the manure pits represents a risk to other farms is important. There is evidence of human enteroviruses surviving and having the ability to percolate into groundwater after the land application of human sewage [12,13,14]. Therefore, the objective of this study was to assess whether PRRSV could percolate through soil.

## 2. Materials and Methods

### 2.1. Virus Selection

For this study, three different type-2 strains of PRRSV from different lineages and restriction fragment length polymorphism (RFLP) patterns, e.g., L1A 1-7-4 (L1A), L1C.5 1-4-4 (L1C.5), and L1G 1-26-2 (L1G), were selected. These strains are currently co-circulating within the U.S. pig population and have been selected given their significant economic impact in swine production systems since their emergence.

### 2.2. Virus Propagation

Viruses were propagated in African green monkey epithelial (MARC-145) cells using Eagle’s minimum essential medium (MEM) (Corning Inc., Corning, NY, USA) supplemented with 8% fetal bovine serum (FBS), neomycin (50 µg/mL), fungizone (1 µg/mL), penicillin (150 IU/mL), and streptomycin (150 µg/mL). The initial titers of these stock viruses were 6.4 log_10_TCID_50_/100 µL for L1A, and 4.75 log_10_TCID_50_/100 µL for L1C.5 and L1G. Prior to adding the viruses to the percolation assessment column model, they were diluted with sterile water (pH of 7.0) to mimic possible field conditions. Diluted titers were 3.83 log_10_TCID_50_/100 µL for L1A, 3.17 log_10_TCID_50_/100 µL for L1C.5, and 3.50 for strain L1G.

### 2.3. Virus Titration

Serial 10-fold dilutions of each PRRSV-2 and percolated virus were prepared in maintenance medium (MEM 4% FBS, neomycin (50 µg/mL), fungizone (1 µg/mL), penicillin (150 IU/mL), and streptomycin (150 µg/mL). All dilutions were inoculated in confluent monolayers of MARC-145 cells (4 wells per dilution). Plates were incubated at 37 °C under 5% CO_2_ and were examined daily under an inverted microscope for the appearance of cytopathic effects (CPE). After 6 days of incubation, virus titers were calculated using the Karber method and were expressed as median tissue culture infectious dose (TCID_50_) per 100 µL [15].

### 2.4. The Soils

A total of 13 Minnesota soil types were obtained, 6 from cropland sites near swine farms and 7 from the Agronomy Department at the University of Minnesota. Soil samples were characterized at the University of Minnesota Soil Testing Laboratory. The following selection criteria were used for the six soil samples obtained from cropland around swine farms: (1) field in the range of 2 miles from the closest swine facility, (2) no manure spreading in at least two years, and (3) not seeded in the current season. Samples were collected in the fall of 2021 according to guidelines from the Department of Soil, Water, and Climate at the University of Minnesota. Briefly, the selected fields were divided into quadrants of approximately 100 m^2^ in a roughly uniform area. Soil samples were collected from 15 points within the pre-established quadrant. For each sampling point, debris was first removed from the surface with a garden shovel, and then a sample of 10 to 15 cm of soil was dug and placed in a bucket. The samples from each sampling point were mixed using the garden shovel and then stored in Ziploc bags at room temperature until being used in the study. Soil samples obtained from the Agronomy Department had been stored under controlled conditions to ensure their compositions and characteristics were not altered over time.

### 2.5. The Percolation Assessment Column Model

**Figure 1:**
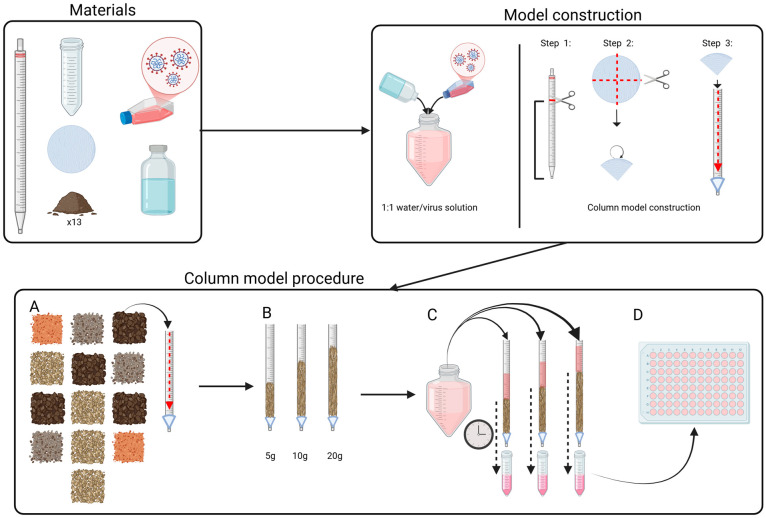
Graphical representation of the column model to assess the ability of PRRSV percolation under experimental conditions.

To assess whether the virus could percolate through soil, a percolation assessment model constructed from 50 mL polystyrene serological pipettes (Stripette™, Corning, NY, USA) was used. The upper end of the serological pipettes was removed for experimental purposes. Filter paper (P4; Fisher Scientific, Hampton, NH, USA) with a 15 cm diameter was cut into four quarters using sterile scissors. Each quarter was folded into a conical structure to fit inside of the serological pipette and placed at the bottom end to contain the soil and allow the free flow of liquid (Figure 1). For each virus, four columns with varying amounts of soil were used: column 1 was a negative control containing filter paper only; column 2 contained 5 g soil and filter paper; column 3 contained 10 g soil and filter paper; and column 4 contained 20 g soil and filter paper.

To standardize soil moisture in the columns and evaluate the time required for water to pass through the soil, sterile water (pH = 7) was added to each column prior to the start of the experiment. Specifically, 3, 5, 10, and 14 mL of water were added to columns 1, 2, 3, and 4, respectively. The percolated virus was collected in 50 mL Falcon^TM^ tubes (Corning, NY, USA) by placing the tube under the tip of the column and waiting until the percolation stopped. The same quantities of diluted PRRSV solution (as those of water, e.g., 3, 5, 10, and 14 mL) were added to the four columns.

Four variables were measured: (a) volume used per column, (b) time (minutes) to first drop, (c) time when percolation stopped, and (d) volume of percolate collected. The percolated solution was mixed by shaking, centrifuged at 2817× *g*, and the supernatant was titrated in triplicates in MARC-145 cells.

### 2.6. Statistical Analysis

A multiple linear regression with initial titer as the fixed effect was carried out to evaluate the differences in virus titer for each PRRSV strain, soil amount, and type of soil. An ANOVA was carried out to evaluate the time of percolation, soil amount, and viral titer for the tested PRRSV strains after verifying that the data met the assumptions of linearity, homoscedasticity, and normality. Analyses were carried out using R version 4.4.1 [16].

## 3. Results

The physicochemical properties of the soils used during the study are summarized in Table 1. Although differences were observed in NO_3_ and phosphorus levels, the electric conductivity and water pH of the sampled soil showed minor variations.

All three PRRSV strains were recovered after percolation, regardless of the amount of soil (5 g, 10 g, and 20 g). However, the amount of soil did affect the virus titer and volume of the percolated dilution. For example, strain L1A was isolated from 13, 12, and 6 of 13 percolates when the amount of soil in the column was 5 g, 10 g, and 20 g, respectively (Table 2). Strain L1C.5 was detected in 13, 6, and 3 percolates (Table 3) while the L1G was isolated from 13, 13, and 11 percolates (Table 4).

Differences in titer were seen after percolation (Table 5) as the L1C.5 titer recovered was statistically significantly lower when compared to the L1G virus (*p* = 0.0134). Similarly, the L1A had a significantly lower titer when compared to the L1G virus (*p* = 0.0428). Additionally, the amounts of soil in the columns also played a role in virus recovery as it showed statistically significant titer reductions (*p* < 0.0001) when comparing either 10 g or 20 g to 5 g. Soils H, I, K, and M had significant differences when compared to soil A (*p* < 0.05). These results suggest that the quantity and type of soil affect viral percolation titers.

**Figure 2:**
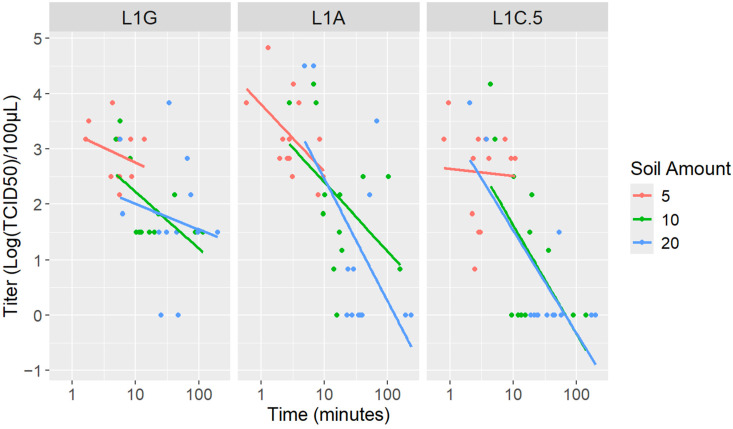
PRRSV strain titer reduction over time of percolation by soil amount.

With higher amounts of soil, a decrease in viral titer and an increase in the time of percolation were observed (Figure 2). Significant differences were detected when assessing time of percolation (*p* < 0.001) and virus (*p* = 0.0002) across treatments; however, there was no statistically significant difference in the soil amount (*p* = 0.5254) (Table 6).

## 4. Discussion

This is the first report of PRRSV percolation through different soils. The observed percolation of the virus in the percolate indicates a potential risk for the virus to migrate through soil. In this study, we used a percolation assessment column model to test the adsorption capabilities of PRRSV through multiple types of soil. Our results indicate that PRRSV can percolate through the soil in the column model regardless of the type and physicochemical properties of the soil evaluated, or the PRRSV strain tested. These findings relate well with other viruses (e.g., poliovirus, echoviruses, coxsackievirus) having the capacity to percolate [17,18,19,20]. Remarkably, some of the percolated dilutions exhibited a higher titer than the positive control, possibly due to variations in the volume of the added virus or a systematic error in the titration process.

Soil factors affecting virus survivability and adsorption are considered to be soil pH, salt species and concentration, organic material, temperature, and moisture content [21]. Virus adsorption is highly associated with pH; 7 to 7.5 pH is optimal, while adsorption decreases with a higher pH [22]. Although in this study there were variations in soil pH, salts, and organic material, they did not seem to affect virus adsorption. Some types of soil and virus strain showed a higher likelihood of percolation than others, but further work is needed to understand factors that affect virus adsorption and percolation. Granting that moisture conditions were standardized for each soil tested, we did not evaluate the effect of temperature.

The presence of PRRSV in swine manure from PRRSV-infected farms and under experimental conditions has been described [11,23,24]. These results indicate the need for further studies to assess the possibility of PRRSV percolation through soils using PRRS-positive manure to mimic cropland fertilizer. The percolation of PRRSV through the different types of soils was not affected by the PRRS strain assessed. However, differences in PRRSV survival among strains have been described, with limitations regarding the methods of the study [25,26]. Thus, virus strain may influence virus survival on cropland after being applied to the soil.

The variability observed between viral strains was surprising given the small physicochemical differences [27]. This might be due to strain-specific properties or, alternatively, systematic error in the column despite the efforts to standardize the procedure, ensuring reproducibility and minimizing bias. These findings do not undermine the conclusions but emphasize the need for further research to understand factors contributing to these variations.

The limitations of this study include the lack of information on PRRSV survivability in water and manure. Our study does not address the issue of virus dilution once it percolates through the soil and contaminates groundwater. However, contamination of groundwater has been addressed, demonstrating concerns about viral contamination of groundwater and the persistence of the virus [13,14,20,26,28,29,30]. Also, our column model does not fully represent field conditions where multiple soil layers exist above the groundwater. Therefore, further studies are needed to elucidate the risk of infecting animals through contaminated water sources including groundwater, and possible control strategies to prevent contaminated water from potentially infecting animals.

## 5. Conclusions

Overall, the PRRSV strains included in this study were capable of percolating through all amounts and types of soil in the column model of this study, regardless of the chemical and physical conditions of soils. Additionally, the PRRSV strain did not seem to be a limiting factor. We demonstrated an inversely proportional relationship between the viral titer and the amount of soil. Further studies are needed to elucidate virus survival in water and the probability of infecting animals through untreated water.

## Figures and Tables

**Table 1:** Physicochemical properties of Minnesota soils used to assess the percolation ability of PRRSV under experimental conditions.

Sample ID	Soil Type	Year	NO3-N	Bray P	Olsen P	NH4OAc-K	LOI OM	Water	1:1 Elec.	Saturation Extract
									Conductivity	Elec. Conductivity
			(mg/kg Soil)	(mg/kg Soil)	(mg/kg Soil)	(mg/kg Soil)	(%)	pH	(mmhos/cm)	(mmhos/cm)
A	Farm Soil	2021	6.84/6.71 *	37/35	0	232/235	4.3/4.2 *	7.2/7.1 *	0.5	-
B	Farm Soil	2021	28.58	99	0	336	4.9	6.3	0.5	-
C	Farm Soil	2021	41.97	81	0	380	4.7	6.8	0.7/0.7 *	-
D	Farm Soil	2021	12.29	52	0	204	4.1	6.5	0.2	-
E	Farm Soil	2021	6.88	54	0	273	4.5	7.1	0.3	-
F	Farm Soil	2021	14.54	78	0	236	5.1	6.4	0.3	-
G	Loam	2019	164.90	76	169/170 *	340	5.0	7.5	1.4	2.8
H	Loamy Sand	2019	6.73	16	0	30	0.5	6.2	0.1	-
I	Silty Clay	2019	200.06	32	0	376	5.2	7.1	1.8	2.4
J	Silty Clay Loan	2019	69.29	31	0	100	2.7	6.4	0.5	-
K	Sandy Clay Loan	2019	85.53	20	0	149	2.0	7.4	0.8	-
L	Sandy Loan	2018	3.14	19	0	61	2.0	5.8	0.1	-
M	Silt Loan	2019	198.41	30	0	229	5.4	6.4	1.2	2.7

* Indicates that samples with two values were analyzed in duplicate to address the variability of the results.

**Table 2:** Evaluation of virus titer, percolated volume, and timing of percolation across soil types and amounts of PRRSV L1A.

Soil ID	Amount of Soil								
	5 g			10 g			20 g		
	Virus Titer in Percolate (log_10_TCID_50_/100)	Percolated Solution (mL)	Time to Percolate Units (hh:mm:ss)	Virus Titer in Percolate Units (log_10_TCID_50_/100)	Percolated Solution (mL)	Time to Percolate (hh:mm:ss)	Virus Titer in Percolate (log_10_TCID_50_/100)	Percolated Solution (mL)	Time to Percolate (hh:mm:ss)
A	2.83	6.2	0:02:49	1.83	12.0	0:09:38	0.00	15.0	0:37:12
B	3.17	5.0	0:02:12	2.17	11.0	0:10:11	0.83	16.0	0:28:51
C	2.50	5.0	0:03:08	0.83	12.0	0:14:08	0.00	14.0	0:34:42
D	2.83	5.7	0:02:36	0.00	12.0	0:15:43	0.00	17.0	0:22:48
E	3.17	6.0	0:02:47	1.5	10.0	0:17:11	0.00	15.0	0:39:41
F	2.83	6.2	0:01:58	2.17	11.0	0:17:39	0.83	16.0	0:23:27
G	2.17	4.7	0:08:03	1.17	13.0	0:18:55	0.00	12.0	0:27:32
H	4.17	5.4	0:03:15	3.83	12.0	0:07:29	3.50	16.0	1:07:23
I	3.83	5.1	0:00:35	4.17	10.0	0:06:43	4.50	14.0	0:04:52
J	3.17	4.3	0:08:29	2.50	8.0	1:43:31	0.00	12.0	3:12:27
K	3.83	5.6	0:03:57	2.50	12.0	0:41:07	2.17	16.0	0:15:43
L	2.50	2.6	0:09:41	0.83	7.0	2:38:00	0.00	11.0	3:56:38
M	4.83	5.8	0:01:17	3.83	11.0	0:02:47	4.50	13.0	0:06:46
C (+)	3.83								

**Table 3:** Evaluation of virus titer, percolated volume, and timing of percolation across soil types and amounts of PRRSV L1C.5.

Soil ID	Amount of Soil								
	5 g			10 g			20 g		
	Virus Titer in Percolate (log_10_TCID_50_/100)	Percolated Solution (mL)	Time to Percolate (hh:mm:ss)	Virus Titer in Percolate (log_10_TCID_50_/100)	Percolated Solution (mL)	Time to Percolate (hh:mm:ss)	Virus Titer in Percolate (log_10_TCID_50_/100)	Percolated Solution (mL)	Time to Percolate (hh:mm:ss)
A	0.83	4.8	0:02:28	0.00	10.0	0:12:02	0.00	14.0	0:24:28
B	3.17	5.2	0:02:47	0.00	11.0	0:09:23	0.00	15.0	0:34:27
C	1.83	5.1	0:02:16	1.50	10.0	0:18:11	0.00	15.0	0:42:20
D	1.50	5.0	0:02:53	0.00	12.0	0:13:31	0.00	15.0	0:21:31
E	1.50	5.0	0:03:01	0.00	9.0	0:21:45	0.00	13.0	0:44:50
F	2.83	5.3	0:02:19	2.17	11.0	0:19:33	0.00	14.0	0:19:03
G	3.17	4.9	0:07:23	0.00	8.0	0:15:28	0.00	13.0	0:24:14
H	3.17	10.0	0:03:44	2.50	10.0	0:10:06	1.50	13.0	0:53:21
I	3.17	4.9	0:00:48	3.17	12.0	0:05:09	3.17	14.0	0:03:46
J	2.83	4.9	0:09:13	0.00	8.0	1:29:23	0.00	16.0	2:53:03
K	2.83	5.0	0:04:07	1.17	11.0	0:35:57	0.00	15.0	0:57:04
L	2.83	7.4	0:10:41	0.00	13.0	2:21:58	0.00	17.5	3:21:56
M	3.83	6.9	0:00:57	4.17	9.0	0:04:24	3.83	10.0	0:02:04
C (+)	3.17								

**Table 4:** Evaluation of virus titer, percolated volume, and timing of percolation across soil types and amounts of PRRSV L1G.

Soil ID	Amount of Soil								
	5 g			10 g			20 g		
	Virus titer in Percolate (log_10_TCID_50_/100)	Percolated Solution (mL)	Time to Percolate (hh:mm:ss)	Virus Titer in Percolate (log_10_TCID_50_/100)	Percolated Solution (mL)	Time to Percolate (hh:mm:ss)	Virus Titer in Percolate (log_10_TCID_50_/100)	Percolated Solution (mL)	Time to Percolate (hh:mm:ss)
A	2.17	4.8	0:05:38	1.50	10.0	0:11:31	3.83	14.0	0:34:19
B	2.50	5.2	0:05:34	1.50	11.0	0:12:24	1.50	15.0	0:31:11
C	2.50	5.1	0:05:30	1.50	10.0	0:19:57	1.50	15.0	0:44:52
D	3.17	5.0	0:05:26	1.50	12.0	0:10:18	0.00	15.0	0:25:16
E	2.50	5.0	0:05:22	1.50	9.0	0:19:48	0.00	13.0	0:47:04
F	3.17	5.3	0:05:18	1.83	11.0	0:23:04	1.50	14.0	0:23:43
G	2.50	4.9	0:08:40	1.50	8.0	0:16:33	1.50	13.0	0:31:07
H	3.83	10.0	0:04:20	2.83	10.0	0:08:16	2.17	13.0	1:14:24
I	3.50	4.9	0:01:50	3.17	12.0	0:04:57	1.83	14.0	0:06:19
J	3.17	4.9	0:08:28	1.50	8.0	1:28:43	1.50	16.0	1:37:42
K	2.50	5.0	0:04:08	2.17	11.0	0:41:22	2.83	15.0	1:05:37
L	3.17	7.4	0:13:45	1.50	13.0	1:57:17	1.50	17.5	3:17:51
M	3.17	6.9	0:01:38	3.50	9.0	0:05:44	3.17	10.0	0:05:43
C (+)	3.50								

**Table 5:** Multiple linear regression of PRRSV titer on percolation assessment model when accounting for amount and type of soil.

	Estimate	SE	95% CI		T-Value	*p*-Value
			Lower	Upper		
Intercept	−0.8484	0.2853	−1.4144	−0.2824	−2.9738	0.0037
L1C.5	−0.4267	0.1695	−0.7629	−0.0904	−2.0515	0.0134
L1A	−0.3477	0.1695	−0.6839	−0.0114	−2.0515	0.0428
Soil amount (10 g)	−1.1708	0.1695	−1.5070	−0.8345	−6.9080	<0.0001
Soil amount (20 g)	−1.6797	0.1695	−2.0160	−1.3435	−9.9111	<0.0001
Soil B	0.2056	0.3528	−0.4944	0.9055	0.5826	0.5614
Soil C	−0.0922	0.3528	−0.7922	0.6077	−0.2614	0.7943
Soil D	−0.4433	0.3528	−1.1433	0.2566	−1.2566	0.2118
Soil E	−0.3133	0.3528	−1.0133	0.3866	−0.8881	0.3766
Soil F	0.4822	0.3528	−0.2177	1.1822	1.3668	0.1747
Soil G	−0.1089	0.3528	−0.8088	0.5911	−0.3086	0.7582
Soil H	1.6122	0.3528	0.9123	2.3122	4.5698	<0.0001
Soil I	1.9467	0.3528	1.2467	2.6466	5.5177	<0.0001
Soil J	0.1867	0.3528	−0.5133	0.8866	0.5291	0.5979
Soil K	0.7789	0.3528	0.0789	1.4788	2.2077	0.0295
Soil L	−0.0733	0.3528	−0.7733	0.6266	−0.2079	0.8358
Soil M	2.4267	0.3528	1.7267	3.1266	6.8783	<0.0001

**Table 6:** ANOVA among PRRSV titers by time of percolation in log (minutes), soil amount, and type of soil.

	Sum sq	Df	F-Value	*p*-Value
Time-Log (minutes)	36.521	1	34.6862	<0.0001
PRRSV strain	17.948	2	8.9828	0.0002
Soil amount	1.293	2	0.6471	0.5254
Residuals	100.891	111		

## Data Availability

Data in this study are contained within the article, and available on request from the corresponding author.
